# Overexpression of the Axl tyrosine kinase receptor in cutaneous SCC-derived cell lines and tumours

**DOI:** 10.1038/sj.bjc.6603135

**Published:** 2006-04-25

**Authors:** J Green, M Ikram, J Vyas, N Patel, C M Proby, L Ghali, I M Leigh, E A O'toole, A Storey

**Affiliations:** 1 Cancer Research UK, Skin Tumour Laboratory, London E1 2AT, UK; 2 Centre for Cutaneous Research, Institute for Cell and Molecular Science, 4 Newark Street, London E1 2AT, UK

**Keywords:** Axl, skin cancer, receptor tyrosine kinase

## Abstract

The molecular mechanisms that underlie the development of squamous cell skin cancers (SSC) are poorly understood. We have used oligonucleotide microarrays to compare the differences in cellular gene expression between a series of keratinocyte cell that mimic disease progression with the aim of identifying genes that may potentially contribute towards squamous cell carcinoma (SCC) progression *in vivo*, and in particular to identify markers that may serve as potential therapeutic targets for SCC treatment. Gene expression differences were corroborated by polymerase chain reaction and Western blotting. We identified Axl, a receptor tyrosine kinase with transforming potential that has also been shown to have a role in cell survival, adhesion and chemotaxis, was upregulated *in vitro* in SCC-derived cells compared to premalignant cells. Extending the investigation to tumour biopsies showed that the Axl protein was overexpressed *in vivo* in a series of SCCs.

Non-melanoma skin cancer (NMSC) is the most prevalent human cancer, with about 1 million cases in the USA and >60 000 cases occurring annually in the UK. Basal cell carcinoma (BCC) is the most common NMSC, whereas squamous cell carcinoma (SCC) accounts for 20% of all cutaneous malignancies. Squamous cell carcinomas typically arise on sun-exposed body sites, and the incidence in Europe, North America and Australia ranges from 50 to 170 cases per 100 000 individuals (Holme *et al*, 2000; Diepgen and Mahler, 2002). Cutaneous SCCs are usually treated by excision; however, they do have the potential to recur locally and, in some cases, to metastasize. Currently, the most common prognostic indicators are histologic subtype and tumour size.

In contrast to BCC development, where mutations in the patched signalling pathway are associated with BCC formation (Johnson *et al*, 1996), the current knowledge on factors that are involved in the *de novo* development and progression of SCC is relatively sparse.

To identify genes that are differentially expressed in SCC compared to normal skin, we have focused in particular on genes encoding cell surface receptors, as overexpression of the encoded protein may serve either as a useful biomarker or a potential target for therapeutic intervention. We have made use of a unique series of cutaneous SCC cell lines derived from an immunosuppressed patient representing different stages of malignant transformation (Proby *et al*, 2000), that serve as a model in which to study the changes in gene expression that occur in the transition from premalignant cells to SCC and finally to a metastatic tumour. They were established from forehead skin (PM1), a primary SCC (MET1) and an associated lymph node metastasis (MET4). Affymetrix GeneChip arrays were used to compare the expression profile of PM1 with two malignant cell lines (MET1 and MET4). Most interestingly, the expression analysis revealed upregulation of Axl, a transmembrane receptor tyrosine kinase with transforming potential (O’Bryan *et al*, 1991) and is an intriguing candidate for involvement in SCC development and progression. Further immunohistochemical analysis of Axl protein expression in a panel of SCCs revealed that Axl was frequently overexpressed in SCCs compared to BCC or normal skin.

## MATERIALS AND METHODS

### Cell culture

Early passage (less than p16) PM1, MET1 and MET4 keratinocyte cell lines were grown with an irradiated fibroblast feeder layer in Dulbecco's modified Eagle's medium plus HAMS F12 medium containing 10% foetal calf serum essentially as described (Rheinwald and Green, 1975).

### Microarray experiments and semi-quantitative reverse transcription–polymerase chain reaction

Briefly, total RNA was extracted from 70–80% confluent PM1, MET1 and MET4 cell lines using TRIZOL Reagent (Invitrogen-15596, Paisley, UK) and was used to generate labelled probes as per manufacturer's instructions. These were used to hybridise the U133A Gene Chips using an Affymetrix Fluidics station 400. Three biological replicates were performed for each of the three cell lines. Full details of the methodology and statistical analysis can be found in Supplementary Material.

Quantitative reverse transcription–polymerase chain reaction (RT—PCR) was performed to validate data from GeneChip experiments and was performed using an OPTICON™ 2 Continuous Fluorescence Detection System (Bio-Rad) on the three replicate samples used in the GeneChip analysis. Full details of the Q–PCR conditions are given in Supplementary Material.

The primer pairs used for amplification of the selected targets were:

Axl: GAGAACATTAGTGCTACGCGGAA/CCTTAGCCCTATGTCCATTAGCA
G6PDH: GTTCCGTGAGGACCAGATCTAC/GGCTCCTTGAAGGTGAGGATAA

### Antibodies and immunohistochemistry

Antibodies used in this study were anti-Axl (C-20), anti-Gas 6 (N-20), (Santa Cruz Biotechnology Inc., Palo Alto, CA, USA), anti-*α* tubulin (Ab-1, Oncogene Science, Cambridge, MA, USA).

Archival paraffin blocks were used for immunohistochemistry; ethical approval for this study was obtained from the East London and City Health Authority Research Ethics Committee.

Axl expression was examined using standard immunohistochemical techniques using 4 *μ*m thick sections that had been deparaffinized, then blocked with rabbit serum and incubated with a goat polyclonal anti-Axl antibody. The sections were then processed as described (Jackson *et al*, 2002). Quantitative analysis of Axl staining was performed using KS400 version 3.0 imaging software (Carl Zeiss Ltd, Welwyn Garden City, UK). The percentage of cells expressing Axl was calculated in four representative high-power fields. Statistical analysis was performed using Arcus Quickstat (Statsdirect, Sale, UK, Biomedical version 1.1) and JMP^©^, SAS Inc. (Karey, NC, USA). For Western blot analysis was performed as described (Jackson *et al*, 2002), signals were detected using ECL+ (Amersham Biosciences UK Ltd, Bucks, UK).

## RESULTS

### Gene expression profiles in premalignant and malignant keratinocyte lines

We compared the relative gene expression levels of the MET1 and MET4 lines with PM1, and also compared expression in MET4 with the solid tumour from which it appears to have originated, MET1 (Popp *et al*, 2000). The profiling revealed that 276 genes were statistically differentially expressed in PM1 cells compared to MET1 and MET4 cells (*P*=0.0001). For practical reasons, we have applied an arbitrary filter level of five-fold changes in the ratio of expression levels; this relatively high cutoff point was used with a view to focus on the genes that are most grossly affected. As a result, an overall comparison of transcript levels from PM1 *vs* MET1, PM1 *vs* MET4 and MET1 *vs* MET4 revealed that 82 genes were significantly differentially expressed with a greater than five-fold change across the three tumour-derived cell lines that fell into diverse functional categories potentially affecting extracellular and intracellular signalling, proliferation and adhesion (Table 1). In particular, we noted that the *axl* tyrosine kinase receptor was significantly overexpressed in the MET1 relative to PM1 cells, and was also overexpressed 4.3-fold in Met4 relative to PM1 cells (Table 1).

### Axl mRNA and protein expression in PM1, MET1 and MET4 cell lines

**Figure 1:**
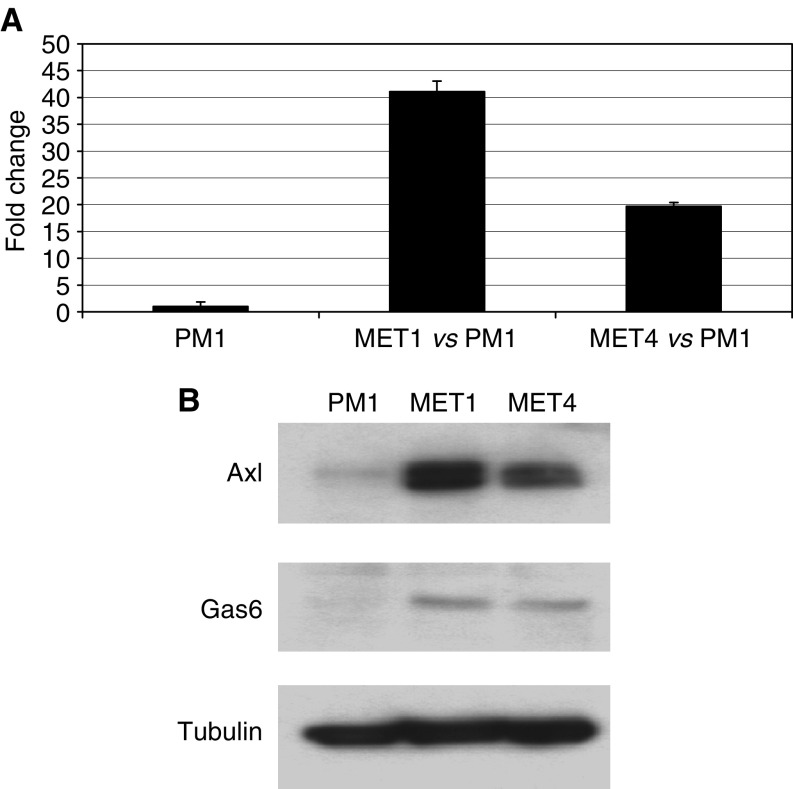
(**A**) Quantitative RT–PCR of *axl* gene expression in PM1, MET1 and MET4 cells. (**B**) Expression of Axl and Gas6, in PM1, MET1 and MET4 cells. Protein extracts were prepared from the different cell lines, separated by SDS–PAGE and Western blotted using specific monoclonal antibodies as described in Materials and Methods. Equal loading of proteins was verified by Western blotting of tubulin.

Quantitative RT–PCR was performed on *axl* transcripts to support the findings of the expression profiling. The analysis was carried out on the RNA prepared for the three biological replicates used in the Affymetrix analysis. The results shown in Figure 1A support the data from the chip analysis. Western blotting of cell lysates showed that Axl protein was also overexpressed in the MET1 and MET4 lines relative to the PM1 line (Figure 1B).

### Immunohistochemical analysis of Axl expression in SCCs

**Figure 2:**
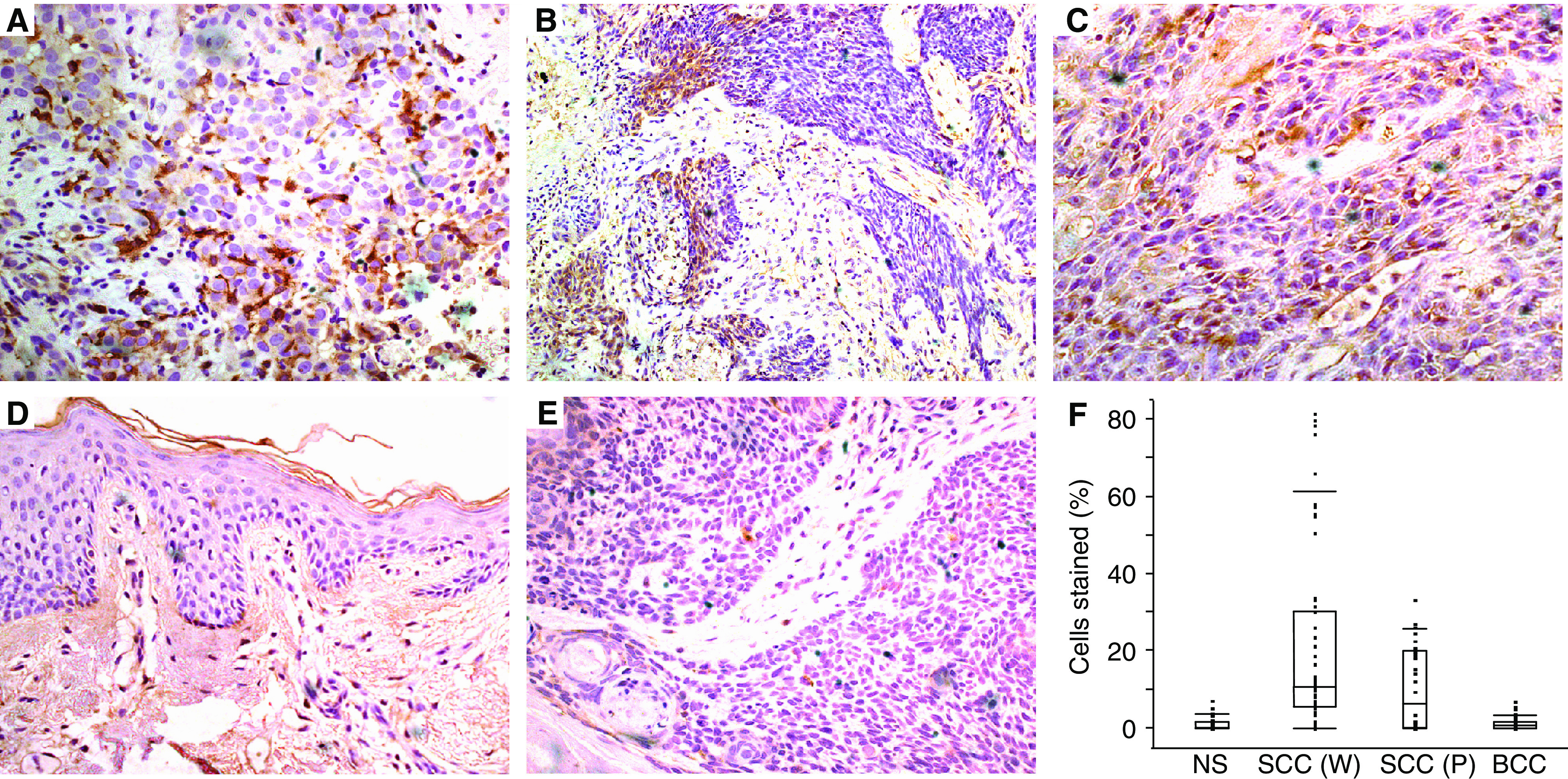
Immunohistochemistry with anti-Axl antibody demonstrates that Axl expression is increased in SCC. (**A**) Membranous and cytoplasmic staining in well-differentiated SCC. (**B**) Heterogeneity of Axl staining in well-differentiated SCC. (**C**) Axl expression in poorly differentiated SCC. (**D**) Axl expression in normal skin. (**E**) Axl expression in BCC. (**F**) Percentage of cells staining with Axl was counted in four high-power fields in each tumour section. The box and whisker plots represent 5th, 25th, 50th, 75th and 95th centiles.

To evaluate the expression of Axl in tumours, we performed an immunohistochemical study on a panel of SCCs, BCCs and normal skin biopsies using anti-Axl-specific antibodies. Axl expression was examined in 17 SCCs (11 well-differentiated and six poorly differentiated) from 16 individuals (Figure 2). Axl expression in 10 BCCs and nine normal skin samples was also investigated. Mast cells that showed consistent, strong, cytoplasmic staining were used in all sections as a positive internal control (data not shown). Goat IgG, at the same concentration as the anti-Axl goat IgG, served as a negative control. Normal epidermis had almost no staining (see Figure 2D) with a mean of 1.3% (95% confidence interval (CI): 0.3 – 2.3) of epidermal cells staining in each section examined. The mean percentage of cells staining with Axl in BCC was 1.3% (95% CI: 0.5 – 2.1%), suggesting that Axl does not have a significant role in cell signalling in BCC (see Figure 2E).

In contrast to normal skin and BCC, 13 out of 17 SCCs (76%) had significant Axl expression. The mean percentage of well-differentiated SCC (SCCW) cells staining with Axl was 21.5 (95% CI: 5.2 – 37.8%). In general, SCC tumour cells exhibited cytoplasmic staining, although there were a few SCC sections where membranous staining of individual cells was detectable (see Figure 2A). Furthermore, one section showed clear heterogeneity in staining within the SCCW (Figure 2B). The poorly differentiated SCC (SCCP) (Figure 2C) group displayed less Axl staining than SCCW, with a mean percentage of cells staining of 10.7% (95% CI: 1.2 – 22.6%). Statistical analysis was performed using Dunnett's Method to compare Axl staining in normal skin and tumours. There was a statistically significant difference between well- and poorly differentiated SCC compared to normal skin (*P*<0.01). There was no significant difference between BCC and normal skin staining with Axl, suggesting that Axl signaling may be restricted to SCCs.

## DISCUSSION

The deregulation of cellular signalling networks underpins much of the basic framework of carcinogenic processes. A striking feature of the expression analysis was the finding that the *axl* gene was greatly upregulated in the MET1 cells compared to PM1. Overexpression of both the mRNA and protein was confirmed in MET1 cells in subsequent experiments. Our results are supported by previous studies in murine SCC where increased *axl* expression was also noted (Loercher *et al*, 2004). For this analysis, we have exclusively focused on genes whose expression was altered more than five-fold. We cannot rule out at this stage that genes whose expression was not changed by more than five-fold between the different cell lines used in this study may play an important role in determining not only the phenotype of these cells, but also in tumour progression *in vivo*.

We then extended the observations on the cell lines to investigate Axl protein expression in a pilot study of SCC biopsies. This analysis showed that Axl expression was increased in a significant proportion of the tumours analysed relative to normal skin. Although this is a small series of tumours, it not only validates the approach of using the PM and MET cell lines as a model system, but also suggests that Axl may be a novel marker whose overexpression is frequently associated with SCC. Whether Axl is also overexpressed in other precursor lesions such as actinic keratoses remains to be investigated. Axl expression was not observed in BCC biopsies, suggesting that Axl is not involved in altering signal transduction pathways in these tumours. *Axl* overexpression has also been noted previously in a variety of other cancers including ovarian (Sun *et al*, 2004), ocular melanoma (van Ginkel *et al*, 2004), osteosacroma (Nakano *et al*, 2003) and renal (Chung *et al*, 2003) tumours. Axl has been shown recently to play an important role in cell migration and proliferation of human endothelial cells (Holland *et al*, 2005). Receptors such as Axl that modulate a number of cellular processes such as growth, adhesion and migration and that are overexpressed on cancer cells, makes them targets for the development of novel therapeutics. Multiple clinical trials have employed novel strategies including antibodies that are antagonistic to such receptors (Finn and Slamon, 2003; Emens and Davidson, 2004; Mineo *et al*, 2004), or alternatively, low molecular weight inhibitors of the kinase activity have also been used, including imatinib mesylate (Gleevec) (Druker, 2004; Pulsipher, 2004; Ross and Hughes, 2004) and gefitinib (Iressa) (Wakeling *et al*, 2002; Blackledge, 2003; Blackledge and Averbuch, 2004; Park *et al*, 2004; Tanno *et al*, 2004). Combination therapy, using both monoclonal antibody together with drug treatment, has also been evaluated (Huang *et al*, 2004). As yet, no specific inhibitors of Axl activity have been described. Our results suggest that further studies aimed at further elucidating the potential role of Axl in SCC are merited, as it may represent a potential therapeutic target for intervention in skin cancer development.

## Figures and Tables

**Table 1:** Gene expression profile using Affymetrix arrays of genes differentially expressed in MET1 and MET4 *vs* PM1cell line and MET1 *vs* MET4.

			**MET1 *vs* PM1**	**MET4 *vs* PM1**	**MET1 *vs* MET4**
**Genbank**	**Common**	**Function**	**Fold +/−**	**Fold +/−**	**Fold +/−**
*Ubiquitin cycle/proteolysis and peptidolysis*					
AA020826	CTSB	Cathepsin B		−5	
		Transport			
N74607	AQP3	Aquaporin 3	−10	−6	
AF201942	NXT2	Nuclear transport factor 2-like export factor 2			8
					
*Transcription/replication*					
NM_004364	CEBPA	CCAAT/enhancer binding protein (C/EBP), alpha	−10		
H94842	HOXA11	Homeo box A11	6	8	
BC005342	NSBP1	Nucleosomal-binding protein 1	16		
NM_016594	FKBP11	FK506-binding protein 11, 19 kDa	5		
AB046692	AOX1	Aldehyde oxidase 1		8	
					
*Signalling*					
NM_006176	NRGN	Neurogranin (protein kinase C substrate, RC3)			11
NM_001945	DTR	Diphtheria toxin receptor)	16	22	
AA464753	PDAP1	Homo sapiens, clone IMAGE:3457786		−8	
M14333	FYN	FYN oncogene related to SRC, FGR, YES	5		
NM_004335	BST2	Bone marrow stromal cell antigen 2			16
NM_013447	EMR2	egf-like, hormone receptor-like sequence 2			−6
NM_003020	SGNE1	Secretory granule, neuroendocrine protein 1			
(7B2 protein)		5			
AI439556	TXNIP	Thioredoxin interacting protein		−5	
					
*Development*					
NM_000366	TPM1	Tropomyosin 1 (alpha)			6
NM_003412	ZIC1	Zic family member 1 (odd-paired homolog, Drosophila)	33	39	
U61276	JAG1	Jagged 1 (Alagille syndrome)		5	
NM_014556	EVC	Ellis van Creveld syndrome			10
					
*Growth factor/chemokine/cytokine/inflammation/immune response*					
NM_002658	PLAU	Plasminogen activator, urokinase	6		
AL573058	C1R	Complement component 1, r subcomponent	13	10	
M92934	CTGF	Connective tissue growth factor			13
NM_003596	TPST1	Tyrosylprotein sulfotransferase 1	8		
NM_013314	BLNK	B-cell linker		6	
NM_002964	S100A8	S100 calcium-binding protein A8 (calgranulin A)	5		
NM_002852	PTX3	Pentaxin-related gene, rapidly induced by IL-1 beta	8		
M69176	CEACAM1	Carcinoembryonic antigen-related cell adhesion molecule 1	6		
NM_006379	SEMA3C	Semaphorin 3C	10		
NM_002053	GBP1	Guanylate-binding protein 1, interferon-inducible, 67 kDa	7		
NM_014391	ANKRD1	Cardiac ankyrin repeat protein	−8		
NM_000963	PTGS2	Prostaglandin-endoperoxide synthase 2	5	16	
L27624	TFPI2	Tissue factor pathway inhibitor 2		9	
BG166705	CXCL5	Chemokine (C-X-C motif) ligand 5		227	
					
*Cell cycle/oncogene/tumour suppressor*					
NM_001759	CCND2	Cyclin D2		−7	8
M15329	IL1A	Interleukin 1, alpha	5		
NM_005504	BCAT1	Branched chain aminotransferase 1, cytosolic	11		
NM_000393	COL5A2	Collagen, type V, alpha 2	44	23	
NM_000584	IL8	Interleukin 8	112	32	
					
*Metabolism/stress response*					
NM_005965	MYLK	Myosin, light polypeptide kinase		−7	7
NM_016816	OAS1	2′,5′-oligoadenylate synthetase 1, 40/46 kDa	5		
AW614435	UROD	Uroporphyrinogen decarboxylase	16		
AV704962	SC4MOL	Sterol-C4-methyl oxidase-like		7	
NM_004265	FADS2	Fatty acid desaturase 2	4	5	
NM_018192	LEPREL1	Leprecan-like 1	10	15	
NM_007034	DNAJB4	DnaJ (Hsp40) homolog, subfamily B, member 4			5
AF054841	TM4SF7	Transmembrane 4 superfamily member 7	−5	−6	
NM_000050	ASS	Argininosuccinate synthetase		−5	
					
*Cytoskeleton/cell adhesion*					
NM_002628	PFN2	Profilin 2	25	15	
U76549	KRT8	Keratin 8		−6	
AI831452	KRT6B	Keratin 6B			5
NM_004791	ITGBL1	Integrin, beta-like 1 (with EGF-like repeat domains)	7		15
AI806793	COL8A2	Collagen, type VIII, alpha 2			8
AI922599	VIM	Vimentin	5		
NM_000422	KRT17	Keratin 17	−5		
					
*Apoptosis*					
NM_006290	TNFAIP3	Tumor necrosis factor, alpha-induced protein 3	7		
BG260394	SNCA	Synuclein, alpha	8	5	
					
*Receptor*					
NM_021913	AXL	AXL receptor tyrosine kinase	12		
NM_000142	FGFR3	Fibroblast growth factor receptor 3		−35	
					
*Others*					
NM_000104	CYP1B1	Cytochrome P450, subfamily I			−7
NM_003118	SPARC	Secreted protein, acidic, cysteine-rich (osteonectin)		−7	
NM_007150	ZNF185	Zinc finger protein 185 (LIM domain)		5	
BF213279	FARP1	FERM, RhoGEF (ARHGEF) pleckstrin domain protein 1	−8		
NM_014903	NAV3	Neuron navigator 3	21	18	
M58026	CALML3	Calmodulin-like 3		−11	
BF217861	MT1E	Metallothionein 1E	15	23	
NM_020672	S100A14	S100 calcium-binding protein A14	−7	−12	
NM_024593	FLJ11767	Hypothetical protein FLJ11767	−6		
NM_000396	CTSK	Cathepsin K (pycnodysostosis)	10	7	
NM_016081	KIAA0992	Palladin	6		
NM_005785	RNF41	Hypothetical SBBI03 protein		6	
NM_004900	APOBEC3B	Apolipoprotein B	8	7	
AF088867	AGR2	Anterior gradient 2 homolog (Xenepus laevis)	32	88	
AF010316	PTGES	Prostaglandin E synthase	−7		−6
NM_002281	KRTHB1	Keratin, hair, basic, 1		−17	
U10691	MAGEA3	Melanoma antigen, family A, 6			−11
AU145365	—	Clone HEMBA1004629, mRNA sequence		7	
AK025430	—	Hypothetical protein FLJ21777		−5	
NM_024554	PGBD5	Piggybac transposable element derived 5	−31		
NM_018436	ALLC	Allantoicase			5
NM_025143	C21orf96	Hypothetical protein FLJ20856		5	
W72694	LOC51063	Hypothetical protein LOC51063		−8	

Only genes whose expression changed by five-fold or more and *P*-value of 0.001 or less are listed.
