# Supplementary material for: Overexpression of the Axl tyrosine kinase receptor in cutaneous SCC-derived cell lines and tumours
Source: Br J Cancer. 2006 Apr 25;94(10):1446–51. doi: 10.1038/sj.bjc.6603135 (PMC2361292; doi:10.1038/sj.bjc.6603135)
Supplement: Supplementary Material [file 94-6603135x1.doc]

**Microarray experiments and quantitative RT PCR**

Total RNA was extracted from 70-80% confluent PM1, MET1 and MET4 cell lines using TRIzol Reagent (Invitrogen-15596, Paisley, UK). Using 5-10μg total RNA, double stranded cDNA was synthesized using Superscript ds-cDNA Synthesis Kit (Invitrogen; 11917-010, Paisley, UK) as per manufacturers instructions and was then cleaned up using Phase Lock Gel (Eppendorf; 0032005 101, Fisher Scientific, Loughborough, UK). This was then used to synthesize cRNA that was labelled using BioArray High Yield RNA Transcript Labeling Kit (ENZO, Affymetrix; 900182, High Wycombe, UK) as per manufacturers instructions. cRNA was purified using RNeasy Mini Kit (QIAGEN; 74104, Crawley, UK) and then quantified. Three biological replicates were performed for each of the three cell lines.

30μg of cRNA was fragmented in fragmentation buffer (40mM Tris-acetate, pH 8.1, 100mM KOAc, 30mM MgOAc) at 94^o^C for 35 minutes. Fragmented and unfragmented cRNA (~μ1g) was electrophoresed on a 1% agarose gel along with a 1kb^+^ DNA marker. Fragmented cRNA generated a smeary band from approximately 35 to 200 bp and unfragmented cRNA produced a smeary band from about 100 bp to 2 kb.

Hybridization Cocktail was made up for human HG_U133 A arrays Gene Chip (Affymetrix 900366) using Eukaryotic Hybridization Control Kit (ENZO, Affymetrix; 900299) 15 μg Fragmented cRNA, 0.05nM Control Oligonucleotide B2, 1x Eukaryotic Hybridization Controls, 0.1 mg/ml Herring sperm DNA, 0.5 mg/ml Acetylated BSA, 1x Hybridization buffer (100 mM MES, 1 M [Na+], 20 mM EDTA, 0.01% Tween 20). The hybridization cocktail was incubated at 99^o^C for 5 minutes and then at 45^o^C for 5 minutes before centrifuging at 1300 rpm for 5 minutes to remove any insoluble material. HG_U133A arrays were equilibrated at room temperature and filled with 200 μl of 1x Hybridization buffer and incubated in oven at 45 ^o^C for 10 minutes with rotation at 60 rpm. Buffer solution was removed from the probe array cartridge, this was then filled with the appropriate volume of the clarified hybridization cocktail and incubated in oven set at 45^o^C with rotation at 60 rpm and hybridized for 16 hours. The GeneChips were washed and stained in the Affymetrix Fluidics station 400 with protocol EukGE_ws2v4 then scanned x 2 in an Affymetrix GeneChip Scanner 2500.

The data was processed using RMA (Gentleman et al., 2004) from within Bioconductor version 1.9.1 (Gentleman et al., 2004) to provide background corrected, quantile normalized data. This normalized data was then imported into GeneSpring^® software version 6.1 program (Silicon Genetics, Redwood City, CA). Each gene was normalized to its median across all samples, and using the cross-gene error model based on replicates within the experimental group to provide a basis for Welch's t-test between the PM1 and MET1, and PM1 and MET4 groups. The data was filtered on the uncorrected p-values, using a threshold of 0.001 (from this 22 false positives were expected in the gene lists).

Quantitative RT-PCR was performed to validate data from GeneChip experiments and was performed using an OPTICON^TM^ 2 Continuous Fluorescence Detection System (Bio-Rad) using the following thermocycler program: 10 minutes of pre-incubation at 95°C followed by 40 cycles for 15 seconds at 95°C and 30 seconds at 60°C. Briefly, cDNA was prepared from 1μg total RNA using the Reverse Transcription System (Promega Corporation, Madison, WI, USA).

Individual real-time PCR reactions were carried out in 20μl volumes containing 1× qPCR MasterMix plus for SYBR Green I ® (EUROGENTEC™), sense and antisense primers for AXL or GAPDH, and 1μl of 1/10 dilution of the cDNA reaction. At the end of the reaction, Cycle threshold (Ct) was manually setup at the exponential level that reflected the best kinetic PCR parameters. Fold increase between PM1/MET1 and PM1/MET2 was calculated as follows: The individual reactions were standardized against the internal control of GAPDH and the average calculated for the triplicates: C(t)Axl-C(t)GAPDH, the difference in Ct between the samples (ΔCt) was used to calculate the fold change. Fold change = 2 ^ΔCt^ using the following thermocycler program: 10 minutes of pre-incubation at 95°C followed by 40 cycles for 15 seconds at 95°C and 30 seconds at 60°C. Briefly, cDNA was prepared from 1μg total RNA using the Reverse Transcription System (Promega Corporation, Madison, WI, USA).
